# Cisplatin-induced hydroxyl radicals mediate pro-survival autophagy in human lung cancer H460 cells

**DOI:** 10.1186/s40659-021-00346-2

**Published:** 2021-07-28

**Authors:** Somruethai Sumkhemthong, Eakachai Prompetchara, Pithi Chanvorachote, Chatchai Chaotham

**Affiliations:** 1 Department of Biochemistry and Microbiology, Faculty of Pharmaceutical Sciences, Chulalongkorn University, 10330 Bangkok, Thailand; 2 Department of Laboratory Medicine, Faculty of Medicine, Chulalongkorn University, 10330 Bangkok, Thailand; 3 Center of Excellence in Vaccine Research and Development (Chula Vaccine Research Center- Chula VRC), Faculty of Medicine, Chulalongkorn University, 10330 Bangkok, Thailand; 4 Department of Pharmacology and Physiology, Faculty of Pharmaceutical Sciences, Chulalongkorn University, 10330 Bangkok, Thailand; 5 Cell-based Drug and Health Products Development Research Unit, Faculty of Pharmaceutical Sciences, Chulalongkorn University, 10330 Bangkok, Thailand

**Keywords:** Cisplatin, Autophagy, Drug resistance, Hydroxyl radicals, Lung cancer

## Abstract

**Background:**

Accumulated evidence demonstrates cisplatin, a recommended chemotherapy, modulating pro-survival autophagic response that contributes to treatment failure in lung cancer patients. However, distinct mechanisms involved in cisplatin-induced autophagy in human lung cancer cells are still unclear.

**Results:**

Herein, role of autophagy in cisplatin resistance was indicated by a decreased cell viability and increased apoptosis in lung cancer H460 cells pre-incubated with wortmannin, an autophagy inhibitor, prior to treatment with 50 µM cisplatin for 24 h. The elevated level of hydroxyl radicals detected via flow-cytometry corresponded to autophagic response, as evidenced by the formation of autophagosomes and autolysosomes in cisplatin-treated cells. Interestingly, apoptosis resistance, autophagosome formation, and the alteration of the autophagic markers, LC3-II/LC3-I and p62, as well as autophagy-regulating proteins Atg7 and Atg3, induced by cisplatin was abrogated by pretreatment of H460 cells with deferoxamine, a specific hydroxyl radical scavenger. The modulations in autophagic response were also indicated in the cells treated with hydroxyl radicals generated via Fenton reaction, and likewise inhibited by pretreatment with deferoxamine.

**Conclusions:**

In summary, the possible role of hydroxyl radicals as a key mediator in the autophagic response to cisplatin treatment, which was firstly revealed in this study would benefit for the further development of novel therapies for lung cancer.

## Background

Cancer is a serious non-communicable disease characterized by the overwhelming growth and proliferation of tumor cells [1]. Among many types, lung cancer contributes the highest incidence and mortality rate in both male and female patients worldwide [2]. Despite the availability of advance therapies, including surgery, radiation, and chemotherapy, the success of lung cancer treatments remains regressive [3]. The combined therapy between anticancer drugs with another treatment has been recommended for lung cancer patients at an advanced stage in order to prolong their survival and improve their quality of life [4]. Nevertheless, the incidence of chemotherapeutic failure and relapse of tumor pathology is continuously being documented. The limited response or resistance to current anticancer drugs is a critical factor that influences treatment failure in lung cancer [5].

Cisplatin is an alkylating agent that has been approved for the treatment of ovarian, bladder, and lung cancers [6]. However, accumulated evidence reveals the resistance to this platinum-containing drug, especially in lung cancer [7–11]. The modulation of apoptosis-regulating proteins is wildly accepted as a major molecular mechanism associated with chemo-resistance. Recently, considerable attention has been paid to the pro-survival autophagic response mediated in cancer cells after exposure to anticancer drugs [12–14]. Although a protective effect of autophagy against cisplatin induced-toxicity has been reported in various cancers, the regulating machinery involved in the autophagic response in cisplatin treated-human lung cancer cells is still unclear [13, 15, 16].

Autophagy is a stress response that conserves homeostasis against environmental changes by recycling cellular components and generating new energy inside the cell [17]. Additionally, autophagy plays an important role in chemotherapeutic resistance in various cancer cells [15–17]. Cancer cells derived from cisplatin treatment highly express an autophagy-regulating protein, Beclin-1, which facilitates the conversion of microtubule-associated protein 1 light chain 3 (LC3-I) to the phosphatidylethanolamine conjugated form (LC3-II), a marker of autophagosome formation [18]. Orchestrate signaling of autophagy begins with the activation of Beclin-1 to initiate the formation of phagophore, a double membrane component, followed by the conjugation between the autophagy-related protein (Atg)12 and Atg5 [19]. To expand the double membrane and construct the autophagosome vesicle, which sequesters damaged cellular organelles/molecules, the Atg12-Atg5 complex together with Atg3 and Atg7 further converts LC3-I to LC3-II. The LC3-II then interacts with p62 (sequestosome 1), an autophagy substrate. Recycling of degraded products from damaged organelles/molecules occurs in the autolysosome vesicle, which arises from the fusion between an autophagosome and a lysosome [20–22].

Various stress stimuli, such as nutrient deprivation, reactive oxygen species (ROS), and damaged proteins/organelles, can activate the autophagic cascade [12]. Because not only DNA damage but also oxidative stress can be generated by cisplatin, the present study aimed to investigate the role of ROS on the pro-survival autophagic response in cisplatin-treated human lung cancer cells. The information gained from this study should enhance the understanding of cisplatin-resistance mechanism and shed light on the further development of an effective chemotherapy for treatment of lung cancer.

## Results

### Cytotoxicity of cisplatin to human lung cancer cells

**Fig. 1:**
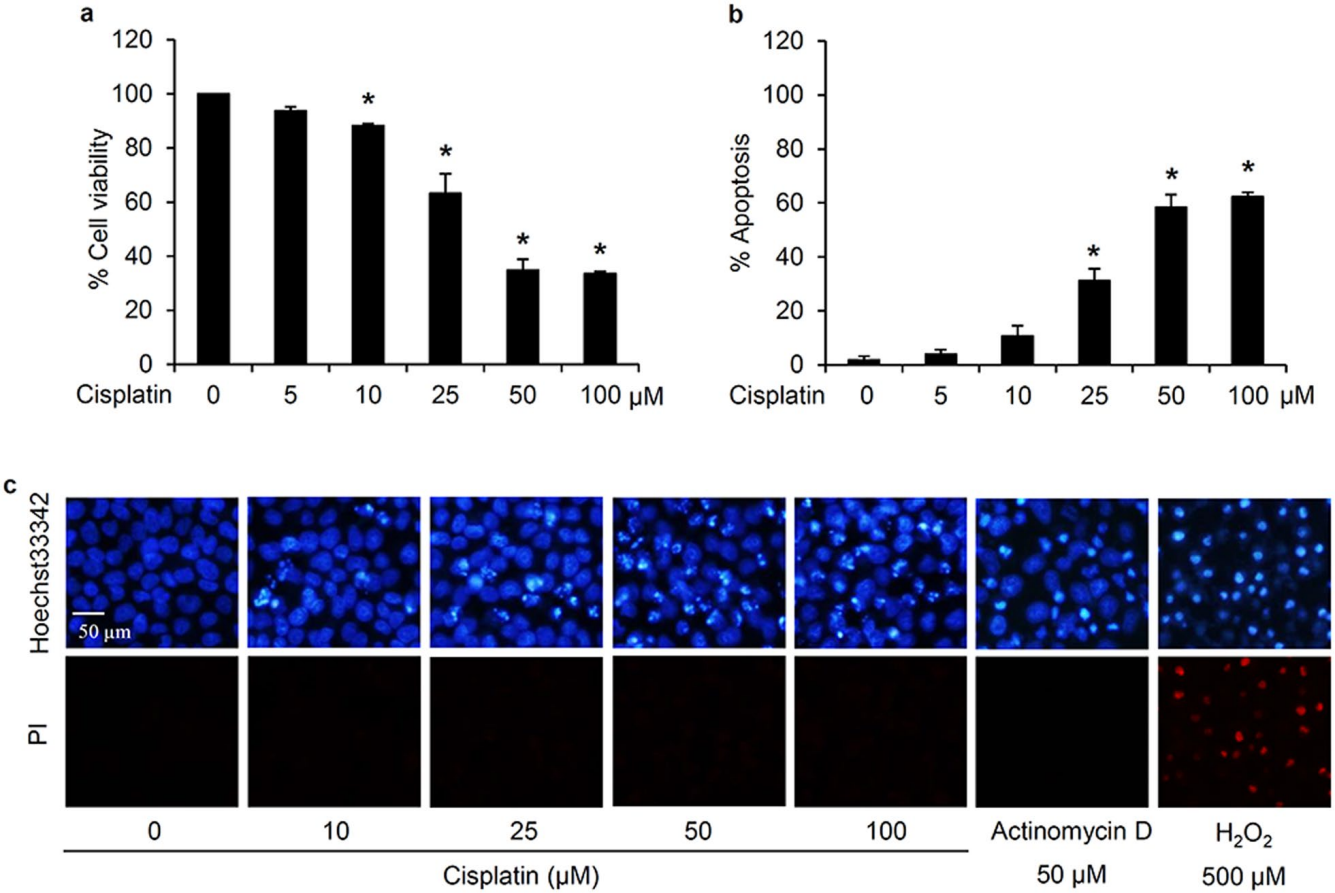
Cisplatin-induced cytotoxicity in human lung cancer cells. **a** MTT assay revealed the significant reduction in the relative cell viability in lung cancer H460 cells after treatment with 10–100 µM cisplatin for 24 h. **b** Increased level of apoptosis was noted after culturing with cisplatin at 25–100 µM. **c** Bright blue fluorescence of Hoechst33342, representing apoptosis, was clearly observed in H460 cells after treatment with cisplatin (25–100 µM) for 24 h, while there was no detectable necrosis (red fluorescence of propidium iodide; PI). Actinomycin D (50 µM) and hydrogen peroxide (H_2_O_2_) at 500 µM was used as a positive control for inducing apoptosis and necrosis, respectively. Data are shown as the mean ± SD (n = 3). *p < 0.05 versus untreated control cells

To clarify the anticancer activity of cisplatin in human lung cancer cells, the relative viability of human lung cancer H460 cells treated with cisplatin at various concentrations was determined via MTT assay. After culture with Roswell Park Memorial Institute (RPMI) medium containing 0–100 µM cisplatin for 24 h, a concentration-dependent reduction in the relative cell viability was observed in the cells incubated with cisplatin at 10–50 µM (Fig. 1a). However, the viability did not further decrease but remained constant at about 40% with the higher cisplatin concentration (100 µM). Mode of cell death was evaluated through nuclear staining assay. Treatment for 24 h with 50 µM Actinomycin D and 500 µM hydrogen peroxide (H_2_O_2_) was respectively used as a positive control for inducing apoptosis and necrosis [23]. Hoechst33342/propidium iodide (PI) costaining demonstrated the presence of apoptotic cells indicating bright blue Hoechst33342 fluorescence of condensed DNA/fragmented nuclei, but not necrotic cells stained with red PI fluorescence, in response to the treatment of H460 cells either with cisplatin (25–100 µM) or Actinomycin D (50 µM) for 24 h (Fig. 1c). In accord with the MTT viability assay, comparable levels of apoptosis were induced by cisplatin at 50 and 100 µM (Fig. 1b). Therefore, 50 µM cisplatin was the lowest concentration that exhibited the maximum cytotoxic activity and was selected for further investigation of the drug resistance mechanisms in human lung cancer cells.

### Cisplatin induces an autophagic response in lung cancer H460 cells

**Fig. 2:**
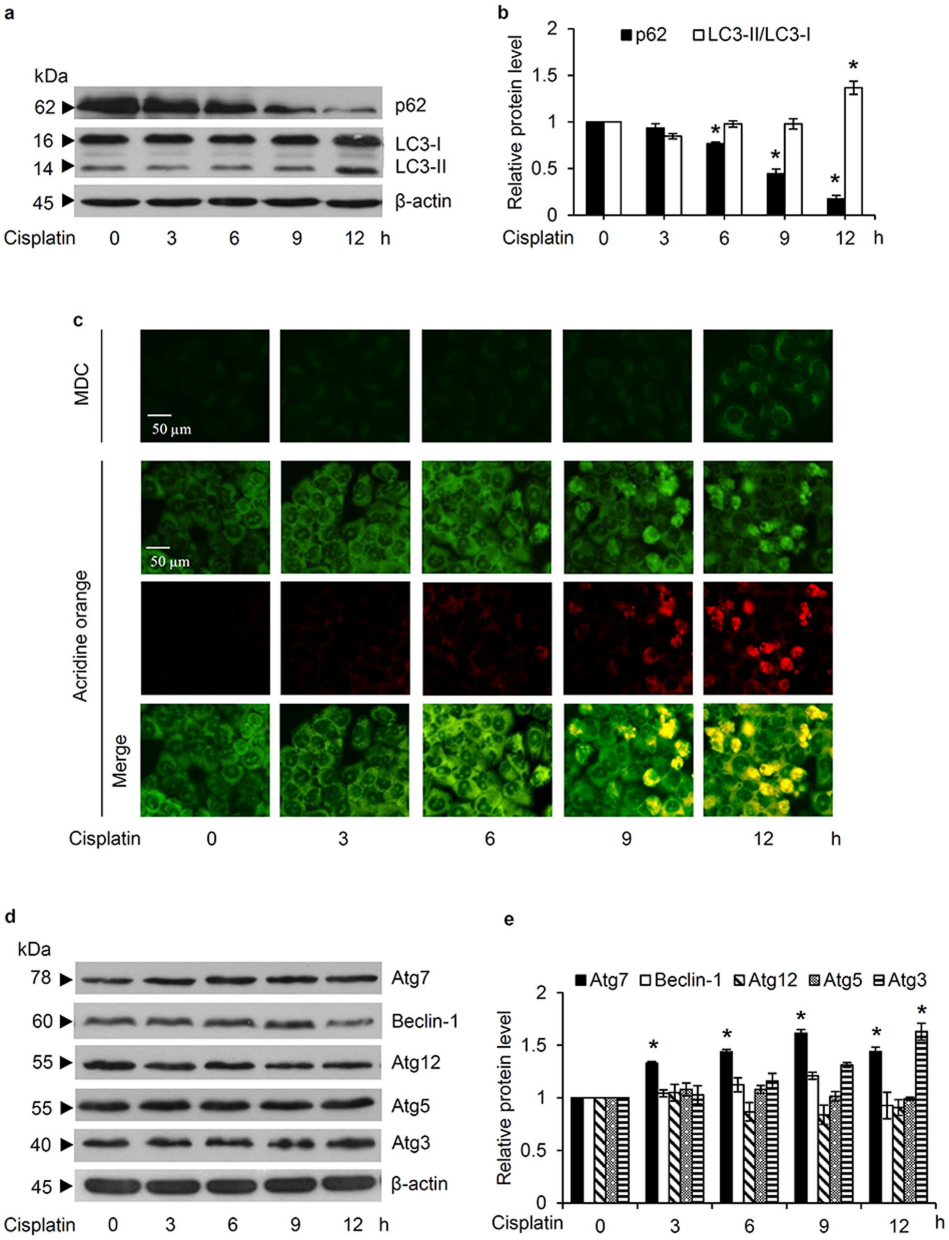
Autophagic response and the alteration of autophagy-related proteins in cisplatin-treated human lung cancer cells. **a** Expression level of the autophagic markers, LC3-II/LC3-I and p62, in H460 cells after culture with 50 µM cisplatin for 0–12 h, as evaluated by western blot analysis. **b** Densitometry analysis indicated that cisplatin up-regulated LC3-II/LC3-I and diminished p62 in a time-dependent manner. **c** Formation of autophagosomes and acidic autolysosomes were observed via the green fluorescence of monodansylcadaverine (MDC) and red fluorescence of acridine orange, respectively, after cisplatin treatment (50 µM). Notably, staining with acridine orange indicates green fluorescence of cytoplasm and nucleolus while bright red/orange-red fluorescence of acridine orange represents acidic compartment. **d** Overexpression of Atg3 and Atg7 in lung cancer H460 cells after 12 h of cisplatin (50 µM) treatment, as revealed by western blot analysis, without (**e**) any significant alteration in Atg5, Atg12, and Beclin-1 expression levels after 0–12 h. Data are shown as the mean ± SD (n = 3). *p < 0.05 versus untreated control cells

To investigate the autophagic response, the alteration of autophagy marker proteins was evaluated in H460 cells cultured with 50 µM cisplatin at various time points (0–12 h). Although western blot analysis revealed a noticeable conversion from LC3-I to LC3-II after 12 h (Fig. 2a), the decreased level of the autophagy substrate, p62, was evident earlier at 6 h of cisplatin treatment (Fig. 2b). Additionally, cisplatin-treated H460 cells were stained with monodansylcadaverine (MDC) in order to evaluate autophagosome formation. Corresponding to the expression level of LC3-II/LC3-I, a slight detection of MDC-labeled vacuoles was indicated in H460 cells cultured with 50 µM cisplatin for 3–9 h and was then strongly observed at 12 h of cisplatin treatment (Fig. 2c). From the different fluorescence color of acridine orange at an acidic and neutral pH, the fusion of a lysosome and autophagosome, which results in acidic autolysosome, can be illustrated with red fluorescence of acridine orange [24]. As demonstrated in Fig. 2c, an accumulation of acidic vacuoles was gradually observed in H460 cells after 6–12 h of the incubation with 50 µM cisplatin. Taken together, these results demonstrated that cisplatin induced an autophagic response in human lung cancer cells in a time-dependent manner.

The underlying mechanisms of cisplatin-induced autophagy in human lung cancer cells were further elucidated. Figure 2d indicates the overexpression of Atg3 and Atg7, the regulatory proteins converting LC3-I to LC3-II, in H460 cells cultured with 50 µM cisplatin for 12 h, which correlated well with the higher expression level of LC3-II/LC3-I at 12 h of cisplatin treatment (Fig. 2a and b). However, the alteration of the other autophagy-regulating proteins (Atg5, Atg12, and Beclin-1) was not significantly detected in human lung cancer cells cultured with 50 µM cisplatin compared to those non-treated control cells (Fig. 2e).

### Autophagy mediates cisplatin resistance in human lung cancer cells

**Fig. 3:**
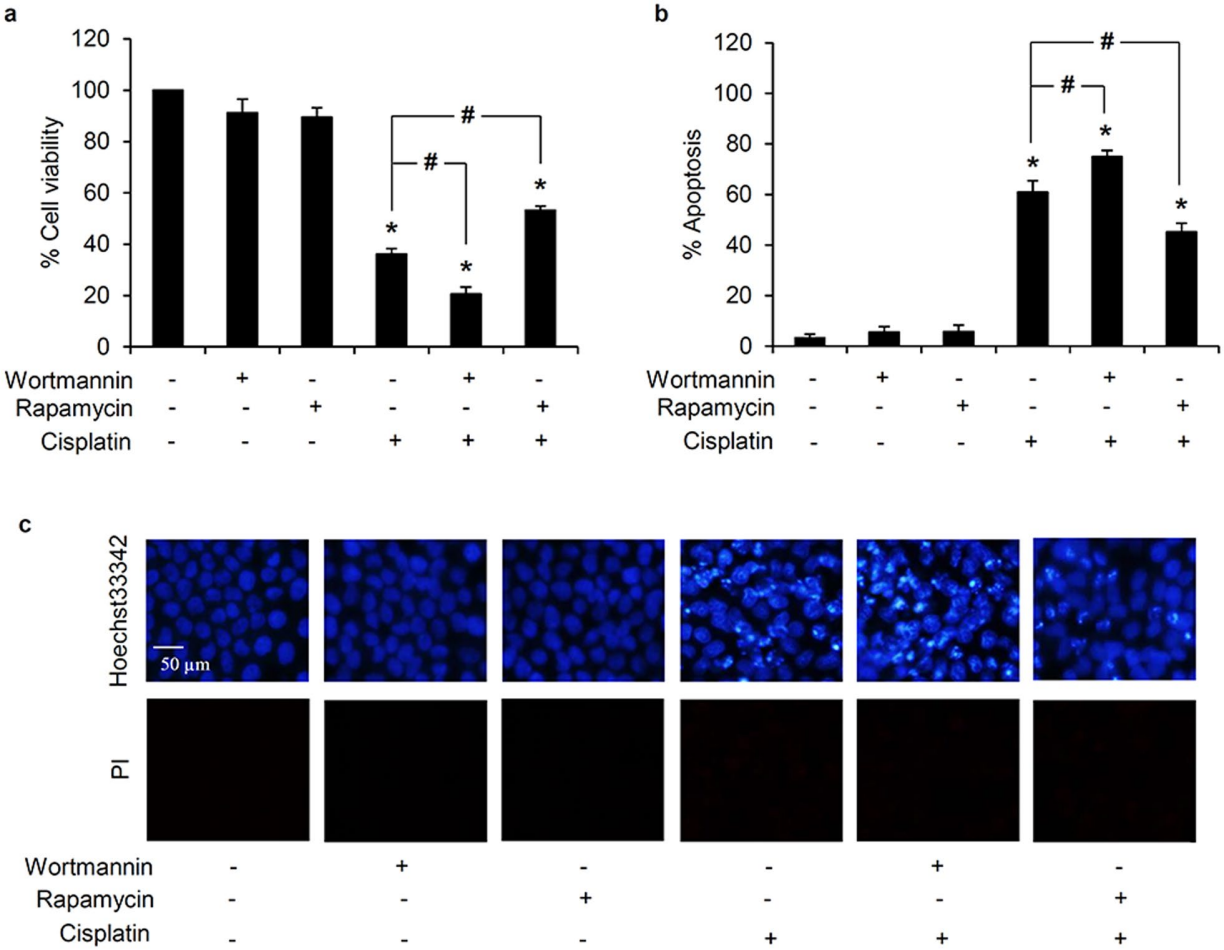
Protective role of autophagy against cisplatin-induced toxicity in human lung cancer cells. **a** Significant alteration of the relative cell viability was observed in H460 cells when pretreated with either 0.5 µM wortmannin (autophagy inhibitor) or 100 nM rapamycin (autophagy inducer) for 30 min followed by 50 µM cisplatin for another 24 h, compared with the cells only treated with cisplatin. **b** The percentage of apoptotic cells demonstrated that the inhibition of autophagy remarkably enhanced cisplatin-induced apoptosis, but (**c**) was inhibited, as evidenced with reduction of bright blue fluorescence of Hoechst33342, by pre-culture with rapamycin. Data are shown as the mean ± SD (n = 3). *p < 0.05 versus untreated control cells. ^#^*p* < 0.05 versus only cisplatin-treated cells

Because the influence of autophagy on chemo-resistance has been frequently reported [13, 15, 16], the protective role of the autophagic response against cisplatin induced-cell death in human lung cancer cells was further confirmed in this study. Lung cancer H460 cells were pre-incubated with either 0.5 µM wortmannin (an autophagy inhibitor) or 100 nM rapamycin (an autophagy stimulator) for 30 min prior to exposure to 50 µM cisplatin. After 24 h of cisplatin treatment, the relative cell viability (Fig. 3a) and level of apoptosis (Fig. 3b) were significantly different in both types of pretreated H460 cells compared with the cells cultured only with cisplatin. As shown in Fig. 3c, pretreatment with the autophagy inhibitor, wortmannin, augmented the level of apoptosis, as evidenced by the condensed DNA and/or fragmented nuclei in H460 cells cultured with 50 µM cisplatin for 24 h. Intriguingly, rapamycin, an autophagy activator, restrained the level of apoptosis in cisplatin-treated H460 cells. These results support the pro-survival role of autophagy on cisplatin resistance in human lung cancer cells.

### Role of hydroxyl radicals on regulation of the autophagic response in cisplatin-treated H460 cells

**Fig. 4:**
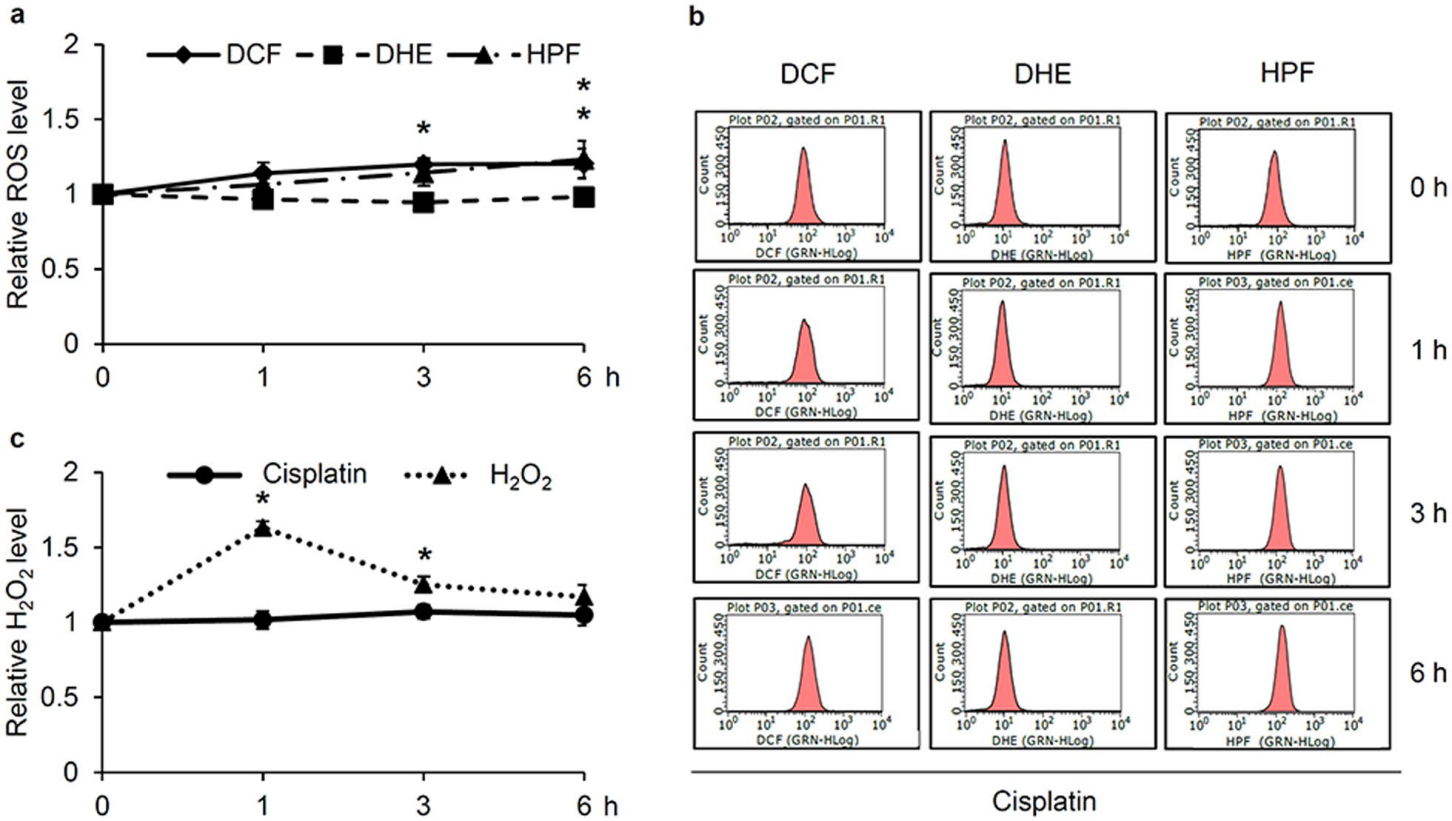
Elevated hydroxyl radicals in cisplatin-treated human lung cancer cells. **a** Augmented level of cellular ROS and hydroxyl radicals (OH^·^) in lung cancer H460 cells after treatment with 50 µM cisplatin for 6 h, as determined by flow cytometric analysis with DCFH_2_-DA and HPF, respectively, **b** without alteration of superoxide anions (O_2_^·−^) levels (flow cytometry chromatogram of DHE). **c** Detection with Amplex red clearly demonstrated the increased relative level of cellular hydrogen peroxide (H_2_O_2_) in H460 cells after culture with 500 µM H_2_O_2_, but not by 50 µM cisplatin. Data are shown as the mean ± SD (n = 3). *p < 0.05 versus untreated control cells

A relationship between intracellular ROS levels and chemotherapeutic resistance has already been documented [25–27]. Moreover, cisplatin has been reported to induce oxidative stress in various cell types [28–30]. Accordingly, the generation of cellular ROS in response to cisplatin treatment of H460 cells was examined in this study. Human lung cancer H460 cells were incubated with 50 µM cisplatin for 0–6 h and then the level of cellular ROS was primary assessed through flow cytometry analysis of the non-specific fluorescence probe, 2′,7′-dichloro-dihydro-fluorescein diacetate ester (DCFH_2_-DA), which interacts with all species of ROS. Figure 4a shows the derived increment in the relative ROS level in H460 cells incubated with cisplatin for 3–6 h. Furthermore, the accumulation of intracellular hydroxyl radicals (OH^·^) was detected using the 3′-(*p*-hydroxyphenyl) fluorescein (HPF) fluorescence probe, with the results showing a time-dependent increase in cisplatin-treated H460 cells and being markedly increased at 6 h of cisplatin treatment. The chromatograms obtained from the flow cytometry indicate a corresponding shift in DCF (total ROS) and HPF (OH^·^) fluorescence intensity in H460 cells cultured with cisplatin for 6 h, while there was no noticeable change of the level of superoxide anions (O_2_^·−^), as presented by the dihydroethidium (DHE) intensity (Fig. 4b). The alteration of the intracellular hydrogen peroxide (H_2_O_2_) level in lung cancer cells cultured with 50 µM cisplatin was determined using the Amplex^®^ Red Hydrogen Peroxide/Peroxidase Assay kit (Life Technologies, Eugene, OR, USA). Incubation with 500 µM H_2_O_2_ for 1 h rapidly increased the relative H_2_O_2_ level (Fig. 4c), as a positive control, whereas with 50 µM cisplatin for 1–6 h did not significantly elevate H_2_O_2_ production in these H460 cells. Thus, most if not all of the ROS formed in cisplatin-treated H460 cells were OH^·^.

**Fig. 5:**
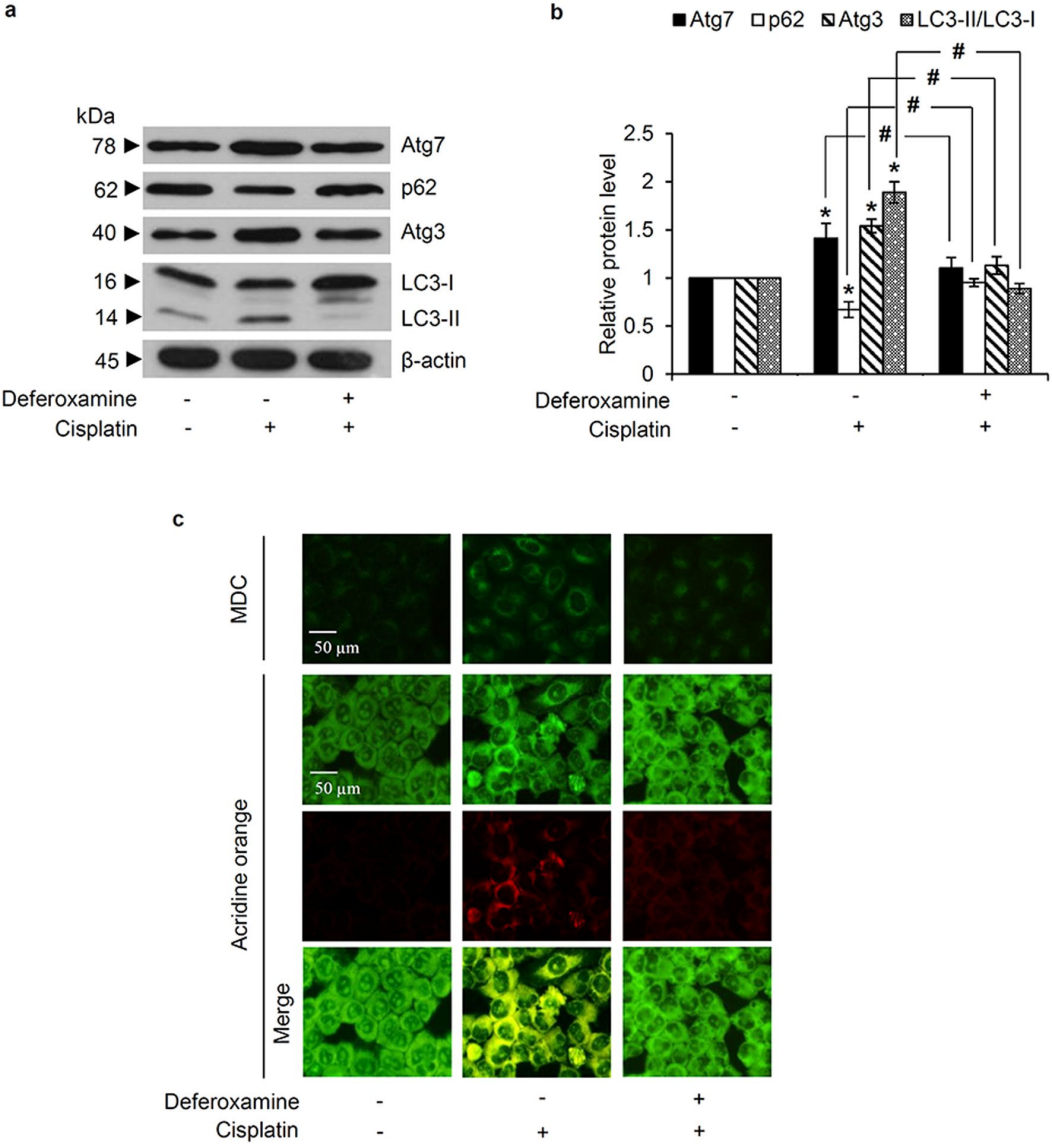
Cisplatin-induced hydroxyl radicals mediate autophagy in human lung cancer cells. **a** Pretreatment with the hydroxyl radical scavenger, deferoxamine (1 mM), reverted the alteration of autophagy-regulating proteins (Atg3 and Atg7) expression levels induced by cisplatin, as evidence by western blot analysis. **b** Significant increment in the LC3-II/LC3-I ratio, and increased Atg3 and Atg7 and decreased expression of p62 by cisplatin treatment was also abrogated by deferoxamine pretreatment. **c** Fluorescence microscopy demonstrated no accumulation of autophagosomes and autolysosomes, as detected by monodansylcadaverine (MDC) and acridine orange staining, respectively, in non-treated control and deferoxamine-pretreated cells. Notably, staining with acridine orange indicates green fluorescence of cytoplasm and nucleolus while bright red/orange-red fluorescence of acridine orange represents acidic compartment. Data are shown as the mean ± SD (n = 3). *p < 0.05 versus untreated control cells. ^#^*p* < 0.05 versus only cisplatin-treated cells

To provide supportive information regarding the role of OH^·^ on the cisplatin-mediated autophagic response, lung cancer H460 cells were pretreated with deferoxamine, a OH^·^ scavenger. After pre-incubation with 1 mM deferoxamine for 30 min, H460 cells were washed with PBS to minimize the direct effect of deferoxamine on autophagy modulation [31, 32] before being cultured with 50 µM cisplatin for 12 h. Pretreatment with deferoxamine significantly suppressed the cisplatin-mediated autophagy, as evidenced by the lower Atg7, Atg3, and LC3-II/LC3-I expression levels in deferoxamine-pretreated H460 cells compared with the cells cultured only with cisplatin (Fig. 5a). Moreover, there was no alteration of the p62 expression level in H460 cells pre-incubated with 1 mM deferoxamine prior to cisplatin, compared with the non-treated control cells (Fig. 5b). The data correspond with the MDC and acridine orange fluorescence dye staining, which revealed the inhibitory effect of the OH˙ scavenger, deferoxamine, on autophagosome and autolysosome formation induced by cisplatin in H460 cells (Fig. 5c).

**Fig. 6:**
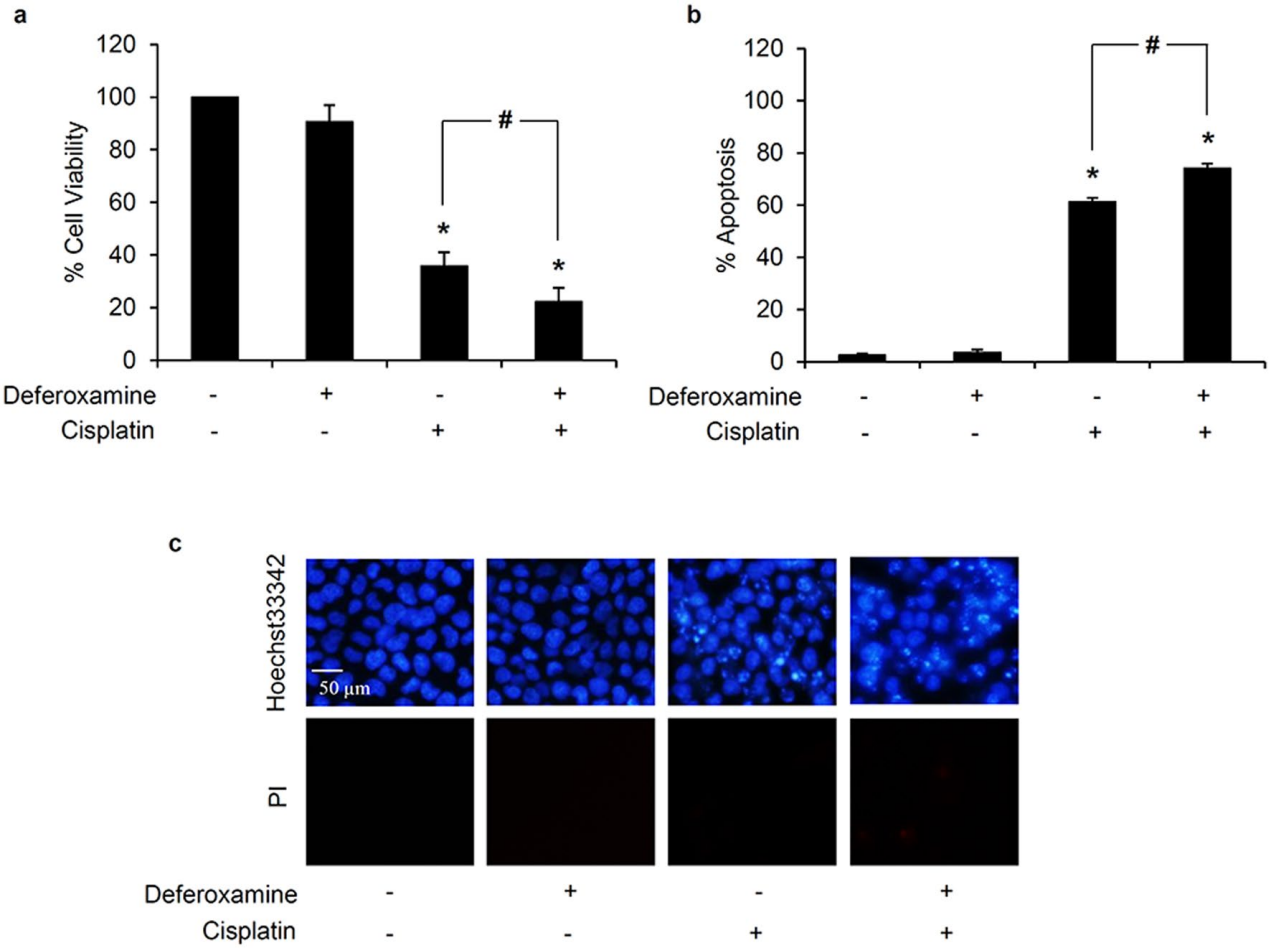
Effect of hydroxyl radical scavenger on cisplatin-induced toxicity in human lung cancer cells. **a** Pretreatment with 1 mM deferoxamine, a hydroxyl radical scavenger, for 30 min prior exposure with 50 µM cisplatin for 24 h significantly reduced cell viability in lung cancer H460 cells compared with the cells only treated with cisplatin. **b** The increment of percentage of apoptotic cells was remarkably observed in H460 cells pretreated with 1 mM deferoxamine in response to cisplatin treatment. **c** Correspondingly, costaining with Hoechst33342/propidium iodide (PI) clearly demonstrated the accumulation of apoptotic cells presenting with bright blue fluorescence of Hoechst33342 without PI red fluorescence-stained necrotic cells in deferoxamine-pretreated H460 cells compared with both untreated control and only cisplatin treated cells. Data are shown as the mean ± SD (n = 3). *p < 0.05 versus untreated control cells. ^#^*p* < 0.05 versus only cisplatin-treated cells

Furthermore, the modulation on cisplatin sensitivity was also determined in deferoxamine-pretreated lung cancer cells. Similarly, human lung cancer H460 cells were pre-incubated with 1 mM deferoxamine for 30 min then the cells were washed with PBS to remove excess deferoxamine and prevent the direct survival activity of deferoxamine [31–33]. After further culture with 50 µM cisplatin for 24 h, the significant reduction of relative cell viability was presented in deferoxamine-pretreated H460 cells compared with the cells only treated with cisplatin (Fig. 6a). Moreover, there was higher %apoptosis (Fig. 6b) assessed via costaining of Hoechst33342/PI (Fig. 6c) in deferoxamine-pretreated H460 cells exposed with cisplatin compared with the cells treated with cisplatin alone. Taken together, these results indicated that cisplatin-induced OH^·^ activate survival autophagic response in lung cancer H460 cells.

### Cellular OH^·^ regulate autophagy in human lung cancer H460 cells

**Fig. 7:**
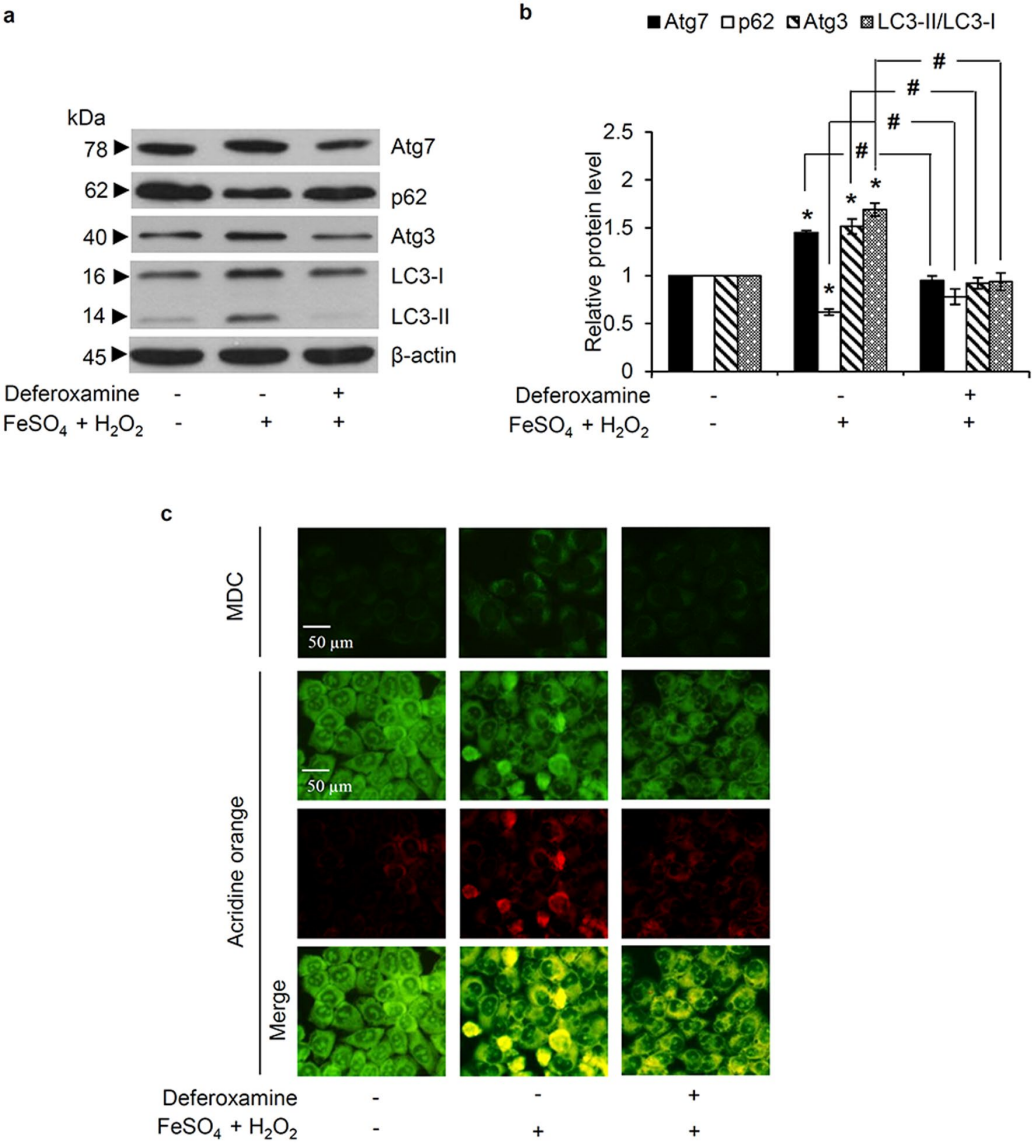
Role of hydroxyl radicals in mediating the autophagic response in human lung cancer cells evidenced with (**a**) up-regulation of LC3-II/LC3-I, Atg3, and Atg7 expression levels, as detected by western blot analysis, after co-culture with 100 µM hydrogen peroxide (H_2_O_2_) and 50 µM FeSO_4_ for 12 h, but (**b**) negated by pre-incubation with 1 mM deferoxamine prior to exposure to H_2_O_2_ and FeSO_4_. (**c**) Autophagosome (green fluorescence of monodansylcadaverine (MDC)) and acidic autolysosome (red fluorescence of acridine orange) formation demonstrate the autophagy stimulating effect of hydroxyl radicals (OH^·^) generated by co-treatment of H_2_O_2_ (100 µM) and FeSO_4_ (50 µM) and its inhibition by deferoxamine pretreatment. Notably, staining with acridine orange indicates green fluorescence of cytoplasm and nucleolus while bright red/orange-red fluorescence of acridine orange represents acidic compartment. Data are shown as the mean ± SD (n = 3). *p < 0.05 versus untreated control cells. ^#^*p* < 0.05 versus H_2_O_2_ + FeSO_4_-treated cells

In order to link the effect of intracellular OH^·^ formation on the autophagic response, an elevated cellular OH^·^ level was generated in H460 cells by co-treatment with H_2_O_2_ (100 µM) and FeSO_4_ (50 µM), known as the Fenton reaction [30]. This resulted in a significant up-regulation of Atg7, Atg3, and LC3-II/LC3-I expression levels (Fig. 7a), while the protein level of p62 was decreased. The role of OH˙ in the regulation of autophagy in H460 cells was further confirmed through pretreatment with 1 mM deferoxamine for 30 min, which was then removed, prior to exposed with co-treatment of H_2_O_2_ and FeSO_4_ to diminish generated cellular OH^·^, the protein levels of Atg7, Atg3, LC3-II/LC3-I, and p62 in the deferoxamine-pretreated cells were not significantly different from the control cells not exposed to the Fenton reaction (Fig. 7b). Likewise, the MDC-labeled autophagosomes and red acridine orange-stained autolysosomes were augmented in H460 cells cultured with H_2_O_2_ and FeSO_4_ for 12 h, while this was abrogated by deferoxamine pre-incubation (Fig. 7c).

## Discussion

To escape from cell death, cancer cells mediate various responsive mechanisms to modulate their survival against chemotherapeutic agents. Among these survival signals, the autophagy has gained in attention [34]. This is a normal cellular defensive mechanism in response to various cellular stresses, such as nutrient deprivation, pathogen infection, and oxidative stress [35], which depends on the lysosomal degradation of damaged cellular organelles, misfolded/aggregated proteins, and harmful stimuli, by which autophagy efficiently promotes cell survival and restrains cell death [16, 36, 37].

Cisplatin-based chemotherapy can prolong the survival of lung cancer patients at an advanced stage [38]. However, there is accumulating evidence of increasing chemo-resistance to this platinum-containing drug in lung cancer cells [7–11]. In order to impede cisplatin-mediated cell death, cancer cells have adopted several drug resistance mechanisms, such as modification of drug uptake/efflux, operation of cellular adduct tolerance, and alteration of apoptosis pathways [39, 40]. Substantial evidence indicates that induction of an autophagic response induced by cisplatin also plays a critical role in the survival and drug resistance in various cancer cells [13, 16, 41].

Likewise, in this study the treatment of lung cancer H460 cells with cisplatin at the maximal response concentration (50 µM) clearly stimulated autophagy, as indicated by the conversion of LC3-I to LC3-II, formation of autophagosomes, and lysosomal degradation of the autophagic substrate, p62 (Fig. 2). Interestingly, pre-culture with an autophagy inhibitor, wortmannin, efficiently sensitized apoptosis in H460 cells treated with 50 µM cisplatin (Fig. 3). These results strongly confirm a positive role for cisplatin-induced pro-survival autophagy in the chemotherapeutic resistance of human lung cancer cells. It should be noted that suppression on autophagic response could also be observed after cisplatin treatment depending on drug susceptibility. In cisplatin-sensitive cancer cells, cisplatin had been reported to mediate apoptosis through inhibition of autophagic survival mechanism [42].

Although DNA intercalation and ROS generation have been well established as cytotoxic mechanisms induced by cisplatin, modulation of the cellular redox status can activate survival signals in cisplatin-treated lung cancer cells [43, 44]. It is worth noting that different species of ROS could be generated by cisplatin depending on the cisplatin concentration and cell type [29, 44, 45]. Detection of ROS with specific fluorescence probes revealed the gradual accumulation of OH˙ in human lung cancer H460 cells cultured with 50 µM cisplatin as early as 6 h, while no alteration of O_2_^·−^ and H_2_O_2_ was noted (Fig. 4).

Oxidative stress has been demonstrated to mediate an autophagic response in different cell types [46–49]. In accord, the elimination of OH˙ through pretreatment with the specific OH^·^ scavenger, deferoxamine, inhibited both the cisplatin- and Fenton reaction-mediated formation of autophagosomes and autolysosomes in human lung cancer cells (Figs. 5c and 7c). In addition, the autophagy markers (LC3-II/LC3-I and p62) and autophagy-regulating proteins (Atg3 and Atg7) were altered by increased cellular OH˙, and abrogated by deferoxamine (Fig. 7a and b). This is the first report about the signaling molecules involving in OH˙-mediating autophagy in human lung cancer cells. On the contrary, modulating on autophagy could in turn alter cellular redox status. As a cellular detoxifying mechanism, suppression of autophagy consequently elevating cellular ROS level is essential for apoptosis induction in lung cancer cells treated with low concentration of cisplatin [50].

In conformity with pro-survival autophagy modulated by OH^·^, resistance to cisplatin-induced cell death, as well as the overexpression of Atg3 and Atg7 in human lung cancer cells cultured with cisplatin was repressed by pre-incubation the cells with deferoxamine (Figs. 5a and b and 6). These support the role of OH˙ as a signaling molecule that triggers an autophagic response in cisplatin-treated lung cancer cells. Cisplatin was previously shown to generate autophagy in cancers through activation of Beclin-1 and Atg5, followed by the conversion of LC3-I to LC3-II [13, 51]. Although, the up-regulated level of Beclin-1, Atg5, and Atg12 were not observed in this study, there was also a significant increase of Atg3 and Atg7, which are essential factors for LC3-II formation, when H460 cells were incubated with cisplatin at 50 µM (Fig. 2d and e). Moreover, the alteration of autophagic markers, autophagosome formation, and fusion of autophagosome-lysosome was sequentially found in the cisplatin-treated H460 cells (Fig. 2). Different regulatory pathways for autophagy in human lung cancer cells might result from diverse concentrations of cisplatin. Focusing on cisplatin-resistant lung cancer cells, the lowest concentration (50 µM) that caused the highest level of apoptosis in the H460 cells was selected in this study, whereas previous studies on cisplatin-mediating autophagy in human lung cancer cells have used concentration at the half maximal inhibitory concentration (IC_50_) or lower [13, 51].

It is the fact that various chemotherapeutic drugs, including doxorubicin have been shown to induce oxidative stress but the suppression on autophagic response is revealed. The accumulation of cellular ROS could be a consequence from the inhibition of autophagosome-lysosome fusion mediated by doxorubicin [52, 53]. Although OH˙ are also generated through redox cycling of doxorubicin [54], whether these free radicals involve with autophagic response after doxorubicin treatment has not been thoroughly investigated.

## Conclusions

**Fig. 8:**
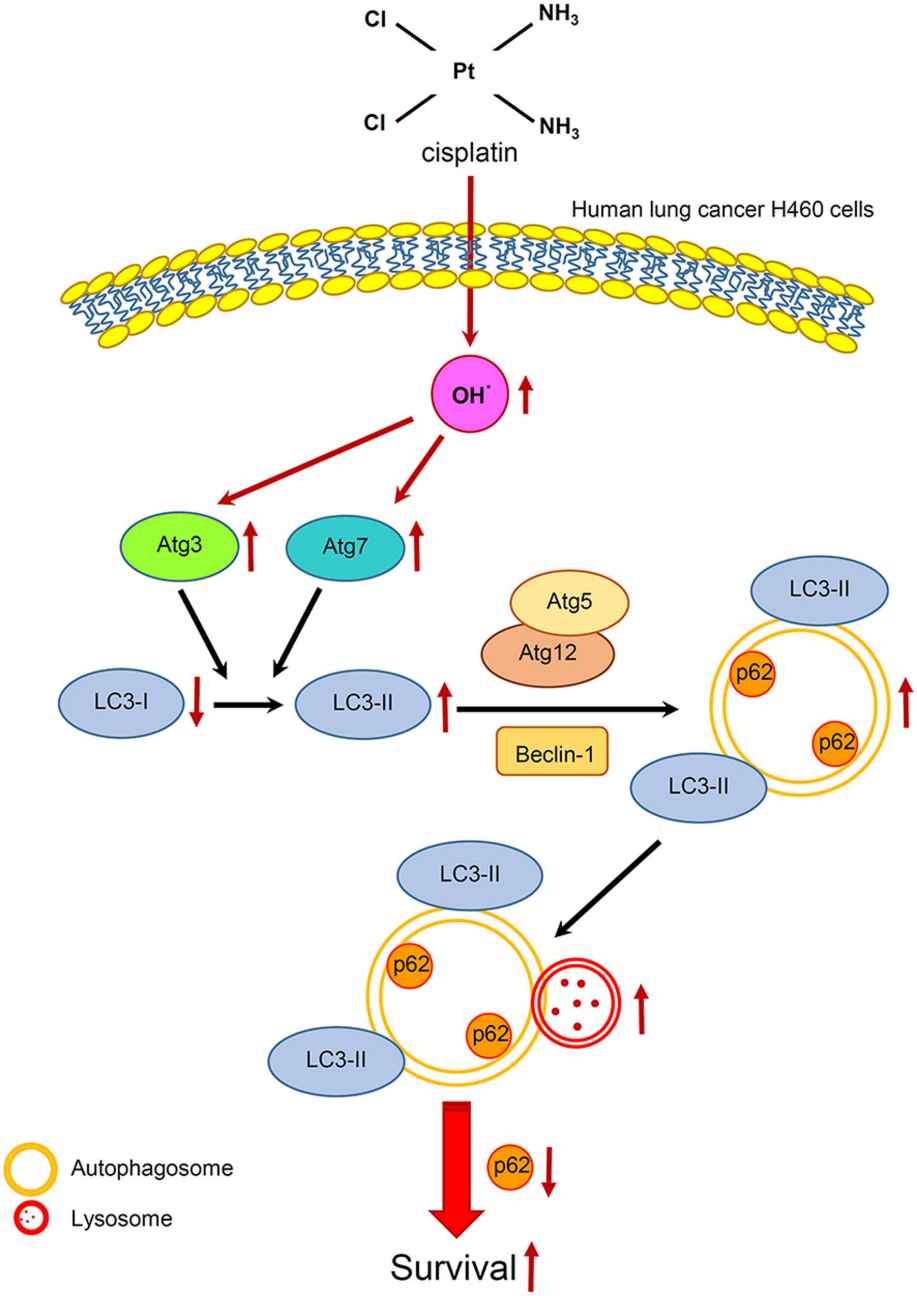
Schematic diagram summarizing the proposed mechanism of cisplatin-induced autophagy in human lung cancer H460 cells. Cisplatin elevates cellular hydroxyl radicals (OH^·^) with consequential activation of Atg3 and Atg7 and conversion of LC3-I to LC3-II. These result in the formation of autophagosomes and reduction of the autophagic substrate p62 through autophagosome-lysosome fusion, and eventually manifest the resistance to cisplatin-induced cell death in human lung cancer H460 cells. Red arrows indicate the modulation on autophagy related molecules induced by cisplatin

The present study indicated that cisplatin at the maximum response concentration mediated autophagy through the induction of OH^·^, which subsequently activated Atg3 and Atg7 and led to apoptosis resistance in human lung cancer H460 cells (Fig. 8). Although the molecular mechanism of cisplatin driving pro-survival autophagy should be further investigated in other lung cancer cells both in in vitro and in vivo experiments, the primary information from this study would benefit for the development of novel strategies for chemotherapeutic treatment, especially in lung cancer.

## Methods

### Chemical reagents

Cisplatin, Actinomycin D, Hoechst33342, propidium iodide (PI), monodansylcadaverine (MDC), acridine orange, 2′,7′-dichloro-dihydro-fluorescein diacetate ester (DCFH_2_-DA), dihydroethidium (DHE), 3′-(*p*-hydroxyphenyl) fluorescein (HPF), deferoxamine, iron (II) sulfate heptahydrate (FeSO_4_·7H_2_O), 30% (w/w) hydrogen peroxide (H_2_O_2_) solution, protease inhibitor cocktail, and skim milk powder were obtained from Sigma Chemical, Inc. (St. Louis, MO, USA). Dimethyl sulfoxide (DMSO) and bovine serum albumin (BSA) were bought from EMD Millipore corporation (Billerica, MA, USA), while 3-(4,5-dimethylthiazol-2-yl)-2,5-diphenyltetrazolium bromide (MTT) and Amplex^®^ Red Hydrogen Peroxide/Peroxidase Assay kit (A22188) were from Life Technologies (Eugene, OR, USA). Wortmannin, rapamycin, primary antibodies against Atg3, Atg5, Atg7, Atg12, Beclin-1, LC3, p62, and β-actin, and specific horseradish peroxidase (HRP)-linked secondary antibodies were acquired from Cell Signaling Technology, Inc. (Danver, MA, USA). Pierce™ Bicinchoninic acid (BCA) protein assay kit and SuperSignal™ West Pico PLUS Chemiluminescent Substrate for western blot analysis were provided by Thermo Fisher Scientific (Waltham, MA, USA).

### Cell culture

Human lung cancer H460 cells (NCI-H460) were obtained from the American Type Culture Collection (ATCC; Manassas, VA, USA). They were grown in Roswell Park Memorial Institute (RPMI) medium supplemented with 10% fetal bovine serum, 2 mM l-glutamine, and 100 units/mL penicillin/streptomycin (Gibco, Life Technologies, NY, USA) in a humidified atmosphere with 5% CO_2_ at 37 °C. Cells at 70–80% confluency (passage 20–40) were used for experiments.

### Cell viability assay

Cell viability was evaluated by the surrogate MTT assay. Briefly, lung cancer cells were seeded at a density of 1 × 10^4^ cells/well in 96-well plates for 12 h. After the indicated treatment, the culture medium was replaced with 0.4 mg/mL of MTT solution and further incubated for 4 h at 37 °C in a dark place. After discarding the MTT solution, DMSO was added to dissolve the purple formazan crystals and the absorbance was read at 570 nm (A_570_) using a microplate reader (Perkin Elmer, Turku, Finland). The relative cell viability (%) was calculated from 100 × the A_570_ ratio between the treated to non-treated control cells.

### Nuclear staining assay

Costaining with Hoechst33342 and PI was performed to evaluate the mode of cell death. After treatment, H460 cells at a density of 1 × 10^4^ cells/well in 96-well plates were incubated with 10 µM Hoechst33342 and 5 µg/mL PI for 30 min at 37 °C. The cells were washed and then observed under an Olympus IX51 inverted fluorescence microscope (Olympus, Tokyo, Japan). Bright blue fluorescence of Hoechst33342 declares chromatin condensation and/or nuclei fragmentation in apoptotic cells, while the red fluorescence of PI indicates necrotic cell death [55]. The relative number of apoptotic cells to the total cell from three different observed areas was calculated and presented as the percentage apoptosis.

### Evaluation of autophagy formation

Human lung cancer H460 cells were incubated with either 0.05 mM MDC or 1 µM acridine orange in phosphate buffer saline (PBS), pH 7.4 (Gibco, Life Technologies, NY, USA) for 15 min at 37 °C in the dark. Before examination under a fluorescence microscope (Olympus IX51, Tokyo, Japan), the staining solution was replaced with PBS. The MDC has been used as a specific dye to detect autophagosome formation [56], while the acidic cellular components of the autolysosome can be identified by the red fluorescence of acridine orange [57].

### Flow cytometry analysis of intracellular ROS

The level of intracellular ROS, O_2_^·−^, and OH^·^ were determined via flow cytometry using the fluorescence probe DCFH_2_-DA, DHE, and HPF, respectively. For this, H460 cells at density of 3 × 10^5^ cells/well were cultured overnight in a 6-well plate, then pre-incubated with 10 µM of either DCFH_2_-DA, DHE, or HPF for 30 min at 4 °C in the dark before further culturing with 50 µM cisplatin for 1–6 h. The cells were then washed and resuspended in PBS prior to immediately analyzing for fluorescence intensity using a Guava easyCyte Benchtop Flow Cytometer (EMD Millipore, Darmstadt, Germany) at an excitation/emission wavelength of 488/538, 488/610, and 490/515 nm for the detection of DCF, DHE, and HPF fluorescence intensity, respectively. The mean fluorescence intensity was quantified using the Guava InCyte version 3.1 software (EMD Millipore). The relative ROS level was derived from the fluorescence intensity ratio at the specific time point to that at 0 h.

### Evaluation of cisplatin-induced hydrogen peroxide in human lung cancer cells

Because of the permeability through the cell membrane, the extracellular H_2_O_2_ released from intracellular compartments can be detected by the specific fluorescence probe, Amplex red (10-acetyl-3,7-dihydroxyphenoxazine) [58–60]. For the assay, human lung cancer cells at a density of 1 × 10^4^ cells/well were cultured in culture medium overnight in a 96-well plate and then cultured with either cisplatin (50 µM) or H_2_O_2_ (500 µM) as a positive control for 30 min. After washing with PBS, the cells were further incubated with 100 µL of reaction mixture (50 µM Amplex red reagent and 0.1 U/mL HRP in Krebs-Ringer phosphate buffer). The fluorescence intensity was determined using a CLARIOstar microplate reader (BMG Labtech, Offenburg, Germany) at an excitation/emission wavelength of 545/590 nm for 1–6 h. The relative H_2_O_2_ level was calculated as the ratio of the fluorescence intensity at the specific time point to that at 0 h.

### Western blot analysis

After the specific treatment, human lung cancer cells were incubated with lysis buffer (20 mM Tris-HCl (pH 7.5), 1% Triton X-100, 150 mM sodium chloride, 10% glycerol, 1 mM sodium orthovanadate, 50 mM sodium fluoride, 100 mM phenylmethylsulfonyl fluoride, and a protease inhibitor cocktail) at 4 °C for 1 h. The total cellular protein of the supernatant collected after centrifugation at 16,000*g* × 15 min (4 °C) was quantitated using a BCA protein assay kit. An equal amount of 35 µg protein from each sample was resolved under denaturing conditions of sodium dodecylsulphate-polyacrylamide gel electrophoresis (12.5% acrylamide resolving gel) and subsequently electro-transferred onto 0.45 µM nitrocellulose membranes (Bio-Rad, Munich, Germany).

The transferred membranes were blocked for 1 h (25 °C) in 5% non-fat dry milk in TBST (25 mM Tris-HCl pH 7.5, 125 mM NaCl, and 0.05% Tween-20) at room temperature and incubated overnight with the specific primary antibody at 4 °C. After washing three times (5 min) with TBST, the membranes were further incubated with the relevant HRP-conjugated secondary antibody for 2 h at 25 °C, and then detected using a chemiluminescence detection kit (Supersignal™ West Pico Plus) and quantified using the analyst/PC densitometry software (Bio-Rad). The intensity of each protein was normalized against that for the β-actin intensity and presented as the relative protein level compared to the non-treated control cells.

### Statistical analysis

All data are presented as the mean ± standard deviation (SD) from three independent experiments. The significance of differences among the groups were evaluated via one-way analysis of variance (ANOVA), followed by Turkey HSD post-hoc test using SPSS version 22. Statistical significance was defined as *p* < 0.05.

## Data Availability

All data generated or analyzed during this study are included in this article.

## Abbreviations

Atg: Autophagy-related protein
BCA: Bicinchoninic acid
BSA: Bovine serum albumin
DCFH_2_-DA: 2′,7′-dichloro-dihydro-fluorescein diacetate ester
DHE: Dihydroethidium
DMSO: Dimethyl sulfoxide
FeSO_4_·7H_2_O: Iron (II) sulfate heptahydrate
HPF: 3′-(*p*-hydroxyphenyl) fluorescein
H_2_O_2_: Hydrogen peroxide
HRP: Horseradish peroxidase
LC3-I: Microtubule-associated protein 1 light chain 3
LC3-II: Phosphatidylethanolamine conjugated form of microtubule-associated protein 1 light chain 3
MDC: Monodansylcadaverine
MTT: 3-(4,5-dimethylthiazol-2-yl)-2,5-diphenyltetrazolium bromide
O_2_^·−^: Superoxide anions
OH^·^: Hydroxyl radicals
p62: Sequestosome 1
PI: Propidium iodide
PBS: Phosphate buffer saline
ROS: Reactive oxygen species
RPMI: Roswell Park Memorial Institute
SD: Standard deviation
